# Supplementary material for: Multimodal CEA-targeted fluorescence and radioguided cytoreductive surgery for peritoneal metastases of colorectal origin
Source: Nat Commun. 2022 May 12;13:2621. doi: 10.1038/s41467-022-29630-9 (PMC9098887; doi:10.1038/s41467-022-29630-9)
Supplement: Supplementary file 1 — Supplementary Information [file 41467_2022_29630_MOESM1_ESM.pdf]

Supplemental material:

S1: Graph of tracer distribution and clearance

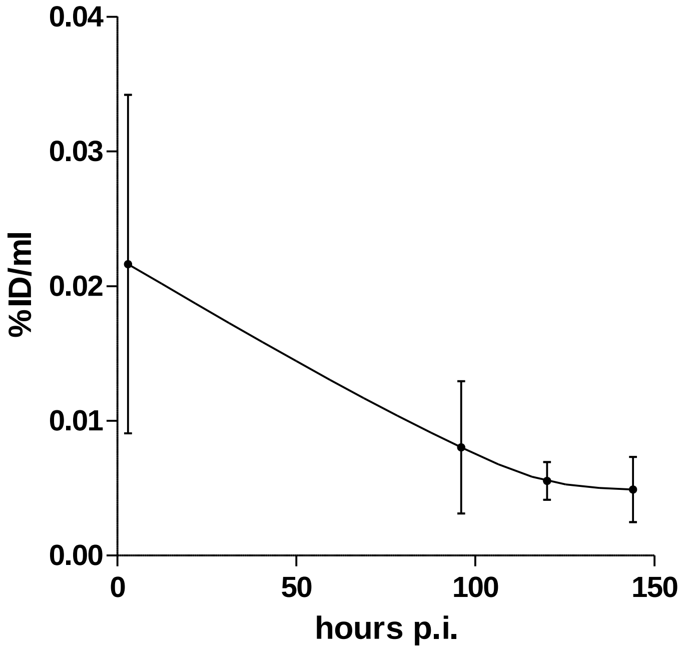

Supplementary figure 1 depict the blood clearance of [^{111}In]In-DOTA-labetuzumab-IRDye800CW over 6 days after injection (p.i.) expressed as mean + SD percentage injected dose per milliliter blood (%ID/ml). N=12 independent cases. 3 cases have been excluded from the analysis because of technical errors resulting in incomplete measurements. Underlying data is reported as a source data file.
