# Supplementary material for: Multimodal CEA-targeted fluorescence and radioguided cytoreductive surgery for peritoneal metastases of colorectal origin
Source: Nat Commun. 2022 May 12;13:2621. doi: 10.1038/s41467-022-29630-9 (PMC9098887; doi:10.1038/s41467-022-29630-9)
Supplement: Supplementary file 3 — Description of Additional Supplementary Files [file 41467_2022_29630_MOESM3_ESM.pdf]

Description of Additional Supplementary Files

File Name: Supplementary Movie 1
Description: Intraoperative imaging reveals peritoneal metastases not detected during visual inspection.
