# Supplementary material for: Multimodal CEA-targeted fluorescence and radioguided cytoreductive surgery for peritoneal metastases of colorectal origin
Source: Nat Commun. 2022 May 12;13:2621. doi: 10.1038/s41467-022-29630-9 (PMC9098887; doi:10.1038/s41467-022-29630-9)
Supplement: Supplementary file 5 — Reporting Summary [file 41467_2022_29630_MOESM5_ESM.pdf]

nature portfolio

Corresponding author(s): Jan Marie de Gooyer

Last updated by author(s): Dec 23, 2021

Reporting Summary

Nature Portfolio wishes to improve the reproducibility of the work that we publish. This form provides structure for consistency and transparency in reporting. For further information on Nature Portfolio policies, see our Editorial Policies and the Editorial Policy Checklist.

Statistics

For all statistical analyses, confirm that the following items are present in the figure legend, table legend, main text, or Methods section.

n/a	Confirmed	
☐	☒	The exact sample size (n) for each experimental group/condition, given as a discrete number and unit of measurement
☐	☒	A statement on whether measurements were taken from distinct samples or whether the same sample was measured repeatedly
☐	☒	The statistical test(s) used AND whether they are one- or two-sided<br>*Only common tests should be described solely by name; describe more complex techniques in the Methods section.*
☒	☐	A description of all covariates tested
☐	☒	A description of any assumptions or corrections, such as tests of normality and adjustment for multiple comparisons
☐	☒	A full description of the statistical parameters including central tendency (e.g. means) or other basic estimates (e.g. regression coefficient) AND variation (e.g. standard deviation) or associated estimates of uncertainty (e.g. confidence intervals)
☐	☒	For null hypothesis testing, the test statistic (e.g. F, t, r) with confidence intervals, effect sizes, degrees of freedom and P value noted<br>*Give P values as exact values whenever suitable.*
☒	☐	For Bayesian analysis, information on the choice of priors and Markov chain Monte Carlo settings
☒	☐	For hierarchical and complex designs, identification of the appropriate level for tests and full reporting of outcomes
☒	☐	Estimates of effect sizes (e.g. Cohen's d, Pearson's r), indicating how they were calculated

Our web collection on statistics for biologists contains articles on many of the points above.

Software and code

Policy information about availability of computer code

Data collection	Clinical data was stored in castor EDC case report farms, CASTOR EDC (2019)
Data analysis	SPSS (version 25 and graphpad prism (version 5.03) was used for data analysis and figure design

For manuscripts utilizing custom algorithms or software that are central to the research but not yet described in published literature, software must be made available to editors and reviewers. We strongly encourage code deposition in a community repository (e.g. GitHub). See the Nature Portfolio guidelines for submitting code & software for further information.

Data

Policy information about availability of data

All manuscripts must include a data availability statement. This statement should provide the following information, where applicable:

- Accession codes, unique identifiers, or web links for publicly available datasets
- A description of any restrictions on data availability
- For clinical datasets or third party data, please ensure that the statement adheres to our policy

All study data are presented in the manuscript and supplementary materials. Underlying data that support figure 4 and supplementary figure 1 are provided as a source data file. Additional raw imaging data that support the findings of this study are available from the corresponding author upon request.

Field-specific reporting

Please select the one below that is the best fit for your research. If you are not sure, read the appropriate sections before making your selection.

☒ Life sciences ☐ Behavioural & social sciences ☐ Ecological, evolutionary & environmental sciences

For a reference copy of the document with all sections, see nature.com/documents/nr-reporting-summary-flat.pdf

Life sciences study design

All studies must disclose on these points even when the disclosure is negative.

Sample size	Because of the exploratory nature of this phase I study, no power calculation was done to determine the sample size. For phase I dose escalation imaging trials these numbers of patients are generally considered sufficient, for example in these two trials conducted by Boogerd et al. & Hekman et al.<br><br>Boogerd LSF, Hoogstins CES, Schaap DP, Kusters M, Handgraaf HJM, van der Valk MJM, Hilling DE, Holman FA, Peeters KCMJ, Mieog JSD, van de Velde CJH, Farina-Sarasqueta A, van Lijnschoten I, Framery B, Pèlegrin A, Gutowski M, Nienhuijs SW, de Hingh IHJT, Nieuwenhuijzen GAP, Rutten HJT, Cailler F, Burggraaf J, Vahrmeijer AL. Safety and effectiveness of SGM-101, a fluorescent antibody targeting carcinoembryonic antigen, for intraoperative detection of colorectal cancer: a dose-escalation pilot study. Lancet Gastroenterol Hepatol. 2018 Mar;3(3):181-191. doi: 10.1016/S2468-1253(17)30395-3. Epub 2018 Jan 30. PMID: 29361435.<br><br>Hekman MC, Rijpkema M, Muselaers CH, Oosterwijk E, Hulsbergen-Van de Kaa CA, Boerman OC, Oyen WJ, Langenhuijsen JF, Mulders PF. Tumor-targeted Dual-modality Imaging to Improve Intraoperative Visualization of Clear Cell Renal Cell Carcinoma: A First in Man Study. Theranostics. 2018 Mar 8;8(8):2161-2170. doi: 10.7150/thno.23335. PMID: 29721070; PMCID: PMC5928878.
Data exclusions	Pharmacokinetic analysis: missing samples (as a result of technical failure or logistic problems) were removed from analysis.
Replication	all labratory measurements were performed in triplicate (pharmacokinetic alicots & lindmo assay)
Randomization	No randomization was performed because of the phase I single arm nature of this study and because a dose escalation strategy was used for safety purposes.
Blinding	Surgeons were blinded to the results of the SPECT/CT scan. Researchers were not blinded but they were not involved in decision making during procedures. Pathologists were blinded for study outcomes.

Reporting for specific materials, systems and methods

We require information from authors about some types of materials, experimental systems and methods used in many studies. Here, indicate whether each material, system or method listed is relevant to your study. If you are not sure if a list item applies to your research, read the appropriate section before selecting a response.

Materials & experimental systems

n/a	Involved in the study	
☐	☒	Antibodies
☒	☐	Eukaryotic cell lines
☒	☐	Palaeontology and archaeology
☒	☐	Animals and other organisms
☐	☒	Human research participants
☐	☒	Clinical data
☒	☐	Dual use research of concern

Methods

n/a	Involved in the study	
☒	☐	ChIP-seq
☒	☐	Flow cytometry
☒	☐	MRI-based neuroimaging

Antibodies

Antibodies used	Labetuzumab (10mg/ml) is a humanized IgG1 monoclonal anti-CEA antibody that was produced and provided by Immunomedics Inc. (Morris Plains, NJ, USA).
Validation	Labetuzumab has been validated as a clinica grade anti-CEA antibody in a variety of previous clinical trials as an antibody and antibody drug conjugate. Our study is the first clinical trial that reports on the dual-labelled version [111In]In-DOTA-labetuzumab-IRDye800CW<br>https://www.cancer.gov/publications/dictionaries/cancer-drug/def/labetuzumab-govitecan?redirect=true<br>https://ncit.nci.nih.gov/ncitbrowser/ConceptReport.jsp?dictionary=NCI%20Thesaurus&code=C95710<br>Hajjar G, Sharkey RM, Burton J, Zhang CH, Yeldell D, Matthies A, Alavi A, Losman MJ, Brenner A, Goldenberg DM. Phase I

radioimmunotherapy trial with iodine-131--labeled humanized MN-14 anti-carcinoembryonic antigen monoclonal antibody in patients with metastatic gastrointestinal and colorectal cancer. Clin Colorectal Cancer. 2002 May;2(1):31-42. doi: 10.3816/CCC.2002.n.009. PMID: 12453334.
Dotan E, Cohen SJ, Starodub AN, Lieu CH, Messersmith WA, Simpson PS, Guarino MJ, Marshall JL, Goldberg RM, Hecht JR, Wegener WA, Sharkey RM, Govindan SV, Goldenberg DM, Berlin JD. Phase I/II Trial of Labetuzumab Govitecan (Anti-CEACAM5/SN-38 Antibody-Drug Conjugate) in Patients With Refractory or Relapsing Metastatic Colorectal Cancer. J Clin Oncol. 2017 Oct 10;35(29):3338-3346. doi: 10.1200/JCO.2017.73.9011. Epub 2017 Aug 17. PMID: 28817371; PMCID: PMC8259133.

Human research participants

Policy information about studies involving human research participants

Population characteristics

The median age of participants was 64 (range 36-73), 4 male and 11 females (not pregnant) All participants were previously diagnosed with a peritoneally metastasized colorectal cancer(adenocarcinoma, mucinous adenocarcinoma or signet ring cell adenocarcinoma).

Recruitment

All patients with peritoneal disease that were deemed eligible for CRS-HIPEC by the surgical oncologist were asked to participate in the study. This could potentially result in selection bias due to inclusion of patients with limited peritoneal disease, and not the one with extensive disease that are not treated with surgery. However, it is not expected that this will have a significant impact on the primary and secondary outcomes in this trial.

Ethics oversight

The study was approved by the regional ethical review board (CMO region Arnhem-Nijmegen) and was performed according to the principles

Note that full information on the approval of the study protocol must also be provided in the manuscript.

Clinical data

Policy information about clinical studies

All manuscripts should comply with the ICMJE guidelines for publication of clinical research and a completed CONSORT checklist must be included with all submissions.

Clinical trial registration

ClinicalTrials.gov NCT03699332

Study protocol

The complete study protocol is accessible to reviewers. It is accessible to third parties in a redacted version upon request.

Data collection

patients were recruited 2 weeks prior to surgery and final data acquisition was completed with 2 months after the surgical procedure for each patient. The first patient was included in December 2018 and the last patient was included in December 2020. Data was collected at the nuclear medicine department (SPECT/CT imaging room, radiochemical laboratorie & radinuclide therapy administration rooms), in the surgical theater & outpatient clinic and at the pathology department.

Outcomes

The primary objectives were to determine the safety and feasibility of multimodal image-guided surgery after intravenous administration of [111In]In-DOTA-labetuzumab-IRDye800CW in patients undergoing CRS-HIPEC for colorectal peritoneal carcinomatosis. Image-guided surgery was considered safe if no serious adverse events were attributed to [111In]In-DOTA-labetuzumab-IRDye800CW administration or study procedures. The study technique was considered feasible when specific accumulation of [111In]In-DOTA-labetuzumab-IRDye800CW in CEA-expressing tumor tissue could be detected with the available technologies (nuclear, optical and (immuno)histological). The optimal protein dose was considered the lowest dose that yielded feasible intraoperative imaging. Secondary outcomes were the number of patients in whom additional lesions were visualized by multimodal imaging after cytoreductive surgery, the radiosignal and fluorescent TBR, correaltion of tracer uptake with pathological assesment and the clearance of the dual labelled tracer. After standard of care surgical resection, fluorescence imaging assisted by radiodetection was used do detect lesions missed by normal visual inspection.
The radiosignalbased TBR was calculated by ex vivo gamma probe measurements of surgical specimens and corresponding benign sections. The fluroescent TBR was calculated by flatbed fluorescence measurements of 4 µm tissue sections containing tumor and benign tissue, which. These sections were also stained for H&E and CEA to correlate tracer accumulation and tumor localisation. Aplicots of the tracer injection were acquired together with blood samples at different timepoints after injection to estimate the blood levels and clearance of [111In]In-DOTA-labetuzumab-IRDye800CW expressed as percentage injected dose per gram (% ID/g).
